# Association of Anatomical Features of the Petrotympanic Fissure and Presence of Foramen of Huschke With Temporomandibular Disorders

**DOI:** 10.1111/joor.13923

**Published:** 2025-01-09

**Authors:** Hacer Eberliköse, Berrin Tuğtağ Demir, Raha Akbarihamed, Hakan Alpay Karasu

**Affiliations:** ^1^ Department of Oral and Maxillofacial Surgery, Faculty of Dentistry Ankara Medipol University Ankara Turkey; ^2^ Anatomy, Faculty of Medicine Ankara Medipol University Ankara Turkey

**Keywords:** cone beam computed tomography, diagnostic imaging, foramen Huschke, petrotympanic fissure, temporomandibular joint disorders

## Abstract

**Background:**

The foramen of Huschke (FH) and the petrotympanic fissure (PTF) are anatomical structures that can influence temporomandibular joint disorders (TMD) by potentially affecting the movement and function of the mandibular condyle.

**Objective:**

This study investigates the relationship between patients with TMD and the presence of FH and PTF to enhance diagnostic and therapeutic approaches.

**Methods:**

This retrospective study analyzed cone beam computed tomography (CBCT) images from 212 patients. Patients were categorised into TMD and control groups based on standardized DC/TMD protocols. An observer, blinded to the patient's clinical status, then analyzed the CBCT images. The CBCT images were evaluated for the presence and characteristics of FH, PTF, and condyle shape and position.

**Results:**

A higher incidence of FH and PTF was observed in patients with TMD than in the control group. FH was present on the right side in 33.3% of patients with TMD and 18% of controls and on the left side in 23.8% of patients with TMD and 10.9% of controls. Open and semi‐open FPT statistically differed between the TMD and control groups (*p* < 0.05). The length of FH in patients with TMD was significantly larger (2.11 ± 0.44 mm) than in the controls (1.67 ± 0.56 mm). The position of the condyle showed a statistically significant difference between the groups (*p* < 0.05).

**Conclusion:**

FH and PTF subtypes are significantly associated with TMD, underscoring their importance in clinical practice.

## Graphical abstract

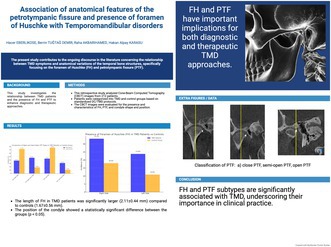

## 1 Introduction

The temporomandibular joint (TMJ), the most complex synovial joint, is closely anatomically related to the ear [1]. The intricate temporal bone contains various crucial structures, including cranial nerves, blood vessels, and the middle ear. Numerous anatomical variations are associated with the TMJ and its disorders. One significant anatomical variation is the foramen of Huschke (FH), located posteromedial to the TMJ in the temporal bone, connecting the external auditory canal (EAC) with the TMJ [2, 3]. FH has been associated with temporomandibular joint disorders (TMD), particularly in cases involving TMJ herniation, with an incidence of 27%, leading to symptoms like otalgia and otorrhea, especially during mastication [4, 5]. However, the precise cause of FH is still not fully understood, but it seems to be associated with factors such as gender, age, ethnicity, and craniofacial abnormalities. Understanding this variation could aid in identifying the origins of complications in the region. A review of the scientific literature revealed that only few studies had investigated the correlation between FH and regional TMJ morphological alterations [3, 6].

Another significant anatomical landmark in the temporal bone is the petrotympanic fissure (PTF), which extends from the TMJ to the tympanic cavity [1]. The PTF connects the middle ear and TMJ and contains the chorda tympani, arteria tympanic anterior, and discomallear ligament. The discomallear ligament connects the TMJ and its capsule to the malleus in the middle ear, passing through the narrow space of the bony PTF. After traversing the PTF, the ligament attaches to the disc or fibers in the posterosuperior section of the TMJ capsule. Therefore, the PTF and the discomallear ligament are crucial for the proper functioning of the TMJ [1]. The configuration of the PTF entry is a vital element in its functional relationship with adjacent anatomical structures, including the condyle of the TMJ. Variations in the morphology of the entrance influence the extent of compression or irritation of structures within the PTF [7].

Understanding these variations and the opening of PTF and FH could help identify the source of complications in the TMJ region, thereby facilitating diagnosis and guiding treatment when necessary. Recurrent infections and local inflammations could alter the bone structures of the TMJ region [5]. Our study is the first to utilize cone beam computed tomography (CBCT) to assess the relationship between the morphology of FH and PTF in patients with temporomandibular disorders, as well as between the shape and position of the mandibular condyle in patients with TMD. The study also investigates the prevalence of FH and PTF. The null hypothesis of our study is that there is no association between the morphology and prevalence of PTF and FH in patients with TMD.

## 2 Material and Methods

### 2.1 Sample and Ethics

This retrospective study was approved by the ethics committee of Ankara Medipol University (ethical no. 111) and complied with the Helsinki Declaration. We conducted a retrospective analysis using CBCT images from patients who visited our clinic between June 2023 and December 2023. The study included 212 patients (414 TMD sides) aged 18–65.

### 2.2 Inclusion Criteria

**FIGURE 1:**
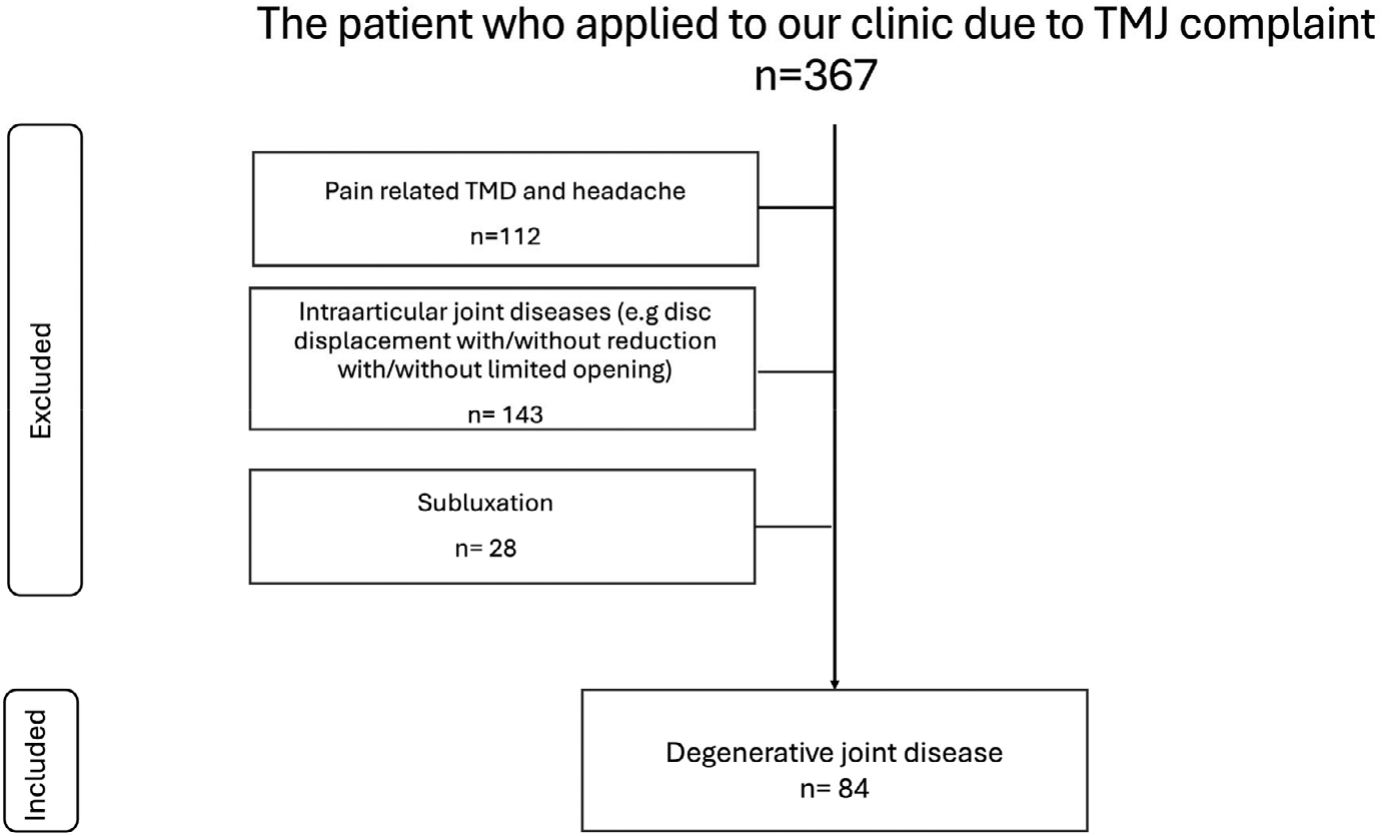
Distribution of patients with temporomandibular disorders (TMD) by subtypes and inclusion criteria.

Participants were categorized into TMD and control groups on the basis of definitive diagnoses according to standardized diagnostic criteria for temporomandibular disorder (DC/TMD) protocols, assessed independently of the imaging analysis. The DC/TMD protocols were employed to determine orofacial pain. Participants selected for the TMD cohort underwent various diagnostic measures, including assessment of TMD‐associated pain and symptoms, collection of demographic data adhering to Axis I criteria, illustration of pain diagrams, involvement in a graded chronic pain scale, application of the Jaw Functional Limitation Scale‐20 and administration of the Patient Health Questionnaire‐9 (PHQ‐9) as delineated under Axis II [8]. The evaluation culminated with the attending practitioner completing a clinical examination form. This protocol includes systematic Axis I and Axis II evaluations, enabling comprehensive classification of TMD subtypes. This study employed to differentiate participants into the following subtypes (Figure 1).

### 2.3 Pain‐Related Temporomandibular Disorders and Headache

Comprises myalgia (local myalgia, myofascial pain, and myofascial pain with referral) arthralgia (TMJ‐related pain), and TMJ‐related headache.

### 2.4 Intra‐Articular Disorders

Disc displacement without reduction (with or without restricted opening).

### 2.5 Degenerative Joint Disease (DJD)

Identified by diminished joint space and abnormalities in cortical bone, confirmed using CBCT imaging.

### 2.6 Subluxation

Characterized by joint hypermobility that impedes the ability to close the mouth from an open position [8].

The criteria for diagnosing DJD were primarily based on cone beam.

Computed tomography (CBCT) imaging findings and due to the high accuracy of CBCT imaging in identifying degenerative joint disorders, we exclusively included patients with verified DJD in our investigation [8].

These criteria include irregularities in the cortical bone around the TMJ, known as an uneven bony cortex [8].

For the control group, patient data were selected from individuals who reported no pain in the TMJ, head, neck or back regions, exhibited no TMJ dysfunction during clinical examination, and had undergone a CBCT for implant therapy or extraction of impacted third molars between June 2023 and December 2023.

Patients with a history of orthodontic or TMJ therapy, those who had trauma, or individuals with congenital craniofacial defects were excluded from the study. Patients were excluded from the study if they had a history of substantial craniofacial trauma, prior TMJ surgeries, or other medical problems that could potentially affect the findings concerning the FH or the PTF. Further exclusions encompassed persons with congenital craniofacial anomalies, systemic illnesses impacting bone metabolism, or incomplete medical records without adequate medical history.

### 2.7 Data Analyses

A maxillofacial surgeon and one anatomist analyzed the CBCT images, blinded to the patient's clinical status (i.e., whether they were part of the TMD or the control group). Each participant was assigned a unique identifier (UID) generated using a secure randomization method.

CBCT scans were obtained while patients were supine. Head alignment was secured using two light beam markers: the vertical marker was aligned with the patients' midsagittal lines to ensure proper centering of the head relative to the rotational axis, and the lateral marker was positioned at the level of the condyles to identify the optimal center of the reconstruction area. Additionally, the head was adjusted so the hard palate was parallel to the floor with the sagittal plane perpendicular to the floor. CBCT scans were performed with 0.5‐mm slices in axial and sagittal planes.

For the CBCT scan analysis, we conducted an in‐depth examination of the PTF topography. Given the high variability of craniofacial bone structure, we utilized the classification system developed by Sato et al. [9]. The CBCT analysis identified Reid's baseline, which is defined in radiology as the line connecting the infraorbital margin and the upper edge of the external auditory meatus. The PTF was then thoroughly examined using an enlarged image in the sagittal plane [1, 10, 11, 12].

**FIGURE 2:**
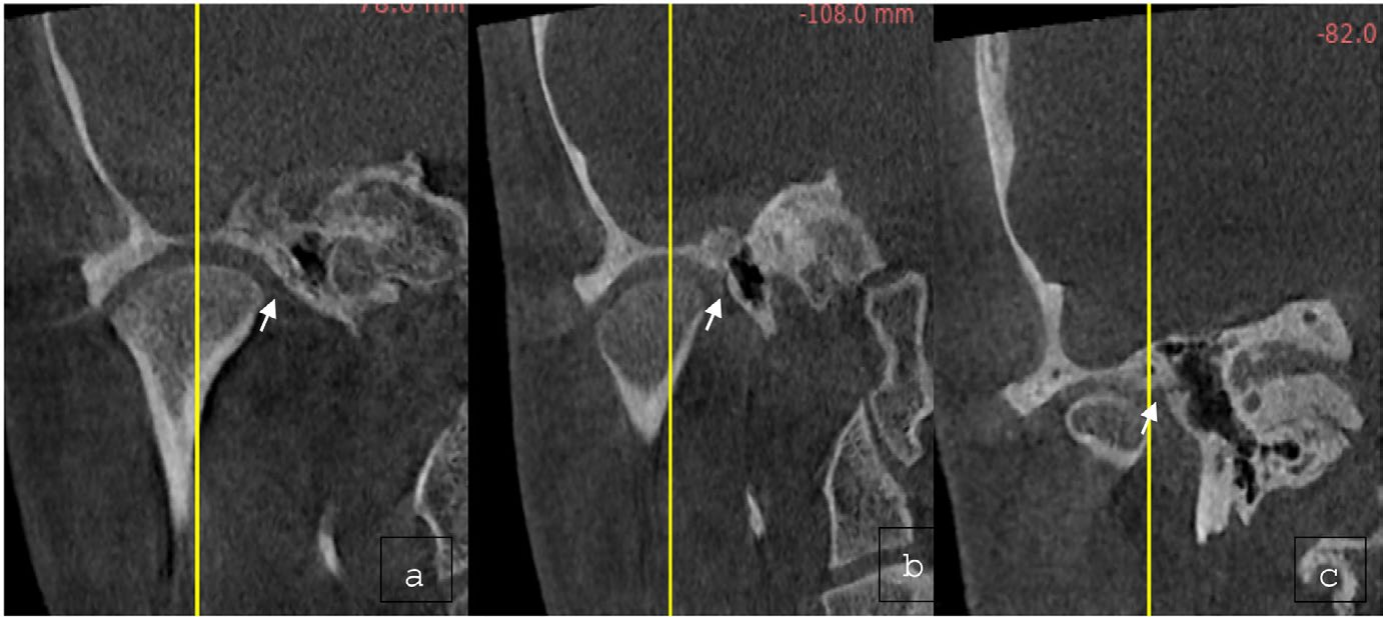
Subtypes of PTF: (a) close PTF, (b) semi‐open PTF, (c) open PTF.

Further analysis revealed that the entrance to the PTF could be classified into three distinct categories. The morphology of the PTF entry is classified into three distinct categories according to its architecture: The open (O) type features a broad and completely unobstructed entrance; the semi‐open (SO) type possesses a partially open entry that is narrower than that of the ‘Open’ type; and the closed (C) type features a securely sealed or nearly obstructed entrance. Subsequently, individual scans of the PTF were analyzed without altering the initial plane positions to ensure reproducibility across cases and were classified as O, SO and C (Figure 2; [7]).

**FIGURE 3:**
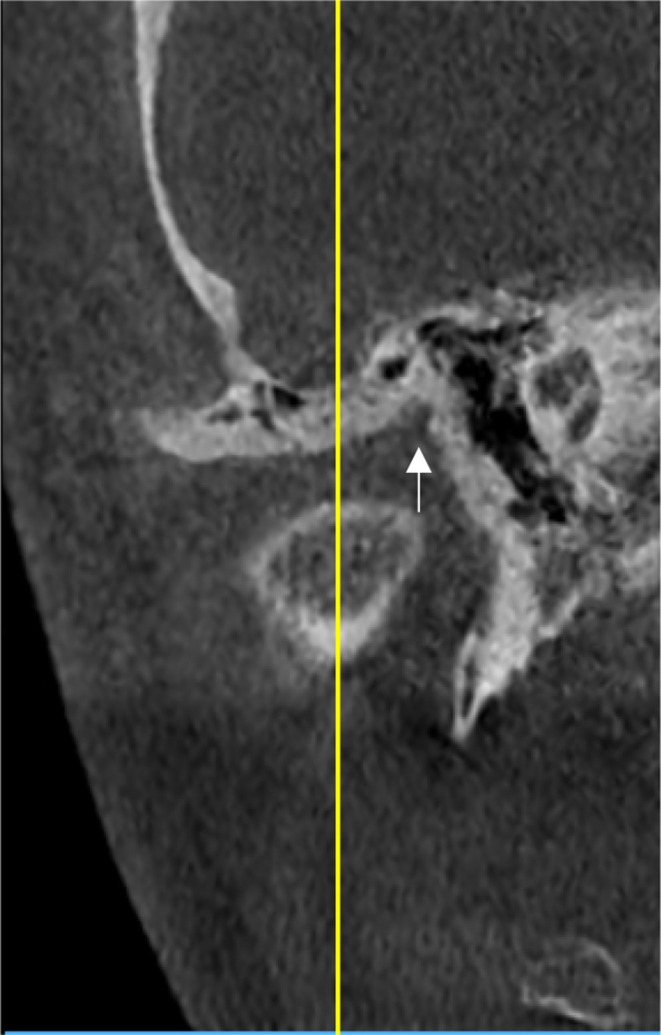
Foramen Huschke in axial slices.

Furthermore, the anteroinferior aspect of the EAC was evaluated with a slice thickness and interval of 1 mm to determine the presence of the FH (Figure 3). After identifying this anatomical structure, its measurement was taken in millimeters at its widest point.

To address our null hypothesis, which assumed a relationship between the prevalence of the PTF and the FH with condylar position and shape, we analyzed the joint's topographical elements in the sagittal plane. Four condylar positions were identified: rear, top‐rear, intracranial‐cranial, and unchanged, with no evidence of displacement [7]. Two types of condylar shapes were identified: straight and oval.

### 2.8 Statistical Analysis

SPSS software (version 12.0.1; SPSS Inc., Chicago, IL, USA) was used to analyze the data. The study examined inter‐observer agreement by calculating intra‐class correlation coefficient. Fisher's exact test was used to compare the foramen prevalence TMD with that of the control group. The chi‐squared test was used to compare the right and left prevalence rates of TMD and Control. The independent *t*‐test was used to compare age means between patients with or without foramen. A confidence interval of 95% was set for the significance of differences (differences were considered statistically significant if their associated *p* < 0.05).

## 3 Results

A total of 212 individuals, comprising 84 patients with TMD and 128 controls, were included in the study, encompassing 424 TMD sides (right and left). The average age of the participants was 37.01 ± 14.13 years (range: 18–79 years), with 51.9% (110) being male and 48.1% (102) being female (Table 1). The frequency of FH between the TMD and control groups demonstrated a statistically significant difference for both the right (*p* = 0.011) and left (*p* = 0.012) sides, with a higher incidence of FH noted in patients with TMD (*p* < 0.05).

**TABLE 1:** PTF types and the prevalence of FH in the TMD and control groups.

			Group		*p*
			TMD	Control	
FH	Right		(33.3%)28_a_	(18.0%) 23_b_	0.010
	Left		(23.8%) 20_a_	(10.9%) 14_b_	0.013
FPT	Right	Open	(38.1%) 32_a_	(72.7%) 93_b_	0.000
		Semi‐open	(46.4.%) 39_a_	(18.0%) 23_b_	
		Closed	(15.5%) 13_a_	(9.4%) 12_a_	
	Left	Open	(44.0%) 37_a_	(76.6%) 98_b_	0.000
		Semi‐open	(31.0%) 26_a_	(10.9%) 14_b_	
		Closed	(25.0%) 21_a_	(12.5%) 16_b_	

*Note:* Test: *x* ^2^, *p* < 0.05, different subscript letters indicate significance.

**FIGURE 4:**
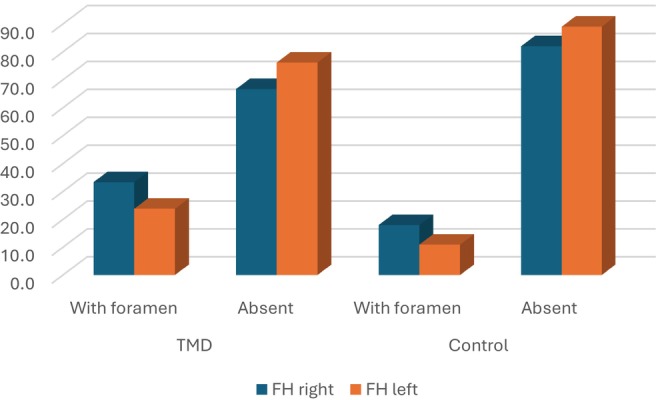
FH distribution in TMD and control groups (in percentage).

FH was found on the right side in 33.3% of patients with TMD and on the left side in 23.8%. In the control group, FH was found on the right side in 18% and on the left side in 10.9% (Figure 4).

The presence of O and SO FPT showed a statistical difference between the TMD and control groups. The rate of O‐type FPT (72.7%) was higher in the control group, and SO‐type FPT (46.4%) was higher in the TMD group (*p* < 0.05) (Table 1).

In patients with FH, the length of the FH was found to be 2.11 ± 0.44 mm in the TMD group and 1.67 ± 0.56 mm in the control group, with the difference being statistically significant (*p* < 0.05). It was observed that the shape of the condyle did not differ significantly between the control and TMD groups (*p* > 0.05); however, the position of the condyle showed a statistically significant difference between the groups (*p* < 0.05) (Table 2).

Inter‐observer agreement was analyzed as 93%.

## 4 Discussion

The present study contributes to the ongoing discourse in the literature concerning the relationship between TMD symptoms and anatomical variations of the temporal bone structures, specifically focusing on the FH and PTF. Our findings indicate a significantly higher incidence of FH and PTF in patients with TMD, thus rejecting the null hypothesis. These anatomical variations have important implications for diagnostic and therapeutic TMD approaches, particularly for maxillofacial surgeons and radiologists.

Previous studies have explored the correlation between these anatomical features and various symptoms associated with TMD. For instance, Çakur and Yaşa [12] observed an increased incidence of tinnitus in patients with TMD with an open pterygomaxillary fissure (PTF). The PTF directly links the auricular cavity and the middle ear. Frequently divided into the petrotympanic and petrosquamous sections, this fissure can serve as a pathway for infectious pathogens to transmit in both directions [13]. Additionally, displacement of anatomical structures in the TMJ can lead to various symptoms related to otological dysfunction. In our study, the SO (right 46.45%; left 31.0%) and C (right 15.5%; left 25.0%) PTF was higher in the TMJ group. Given that fissures of the C type are long and narrow, we hypothesized that it is unlikely for microcirculation to develop within the vessels of these fissures. Our results support a strong correlation between the location and shape of the PTF entrance and the displacement of the rear condyle. Similarly, Skog et al. [14] reported that tinnitus was higher in patients with TMJ symptoms than the healthy population. Contrary to these findings, our study found a higher prevalence of open PTFs in the healthy group, whereas most SO PTFs were detected in the right TMD group. We hypothesize that the discrepancy may be attributed to the discomallear ligament within the PTF, which connects the malleus in the ear to the TMJ disc. The pathological closure of this anatomical linkage could potentially restrict the movements of the TMJ disc, contributing to TMJ symptoms. Hence, the status of PTF can be considered a significant factor in the etiology of TMJ. Further investigations, including detailed soft tissue analysis or clinical correlation with internal derangement, would be beneficial to elucidate the relationship between these structures.

In the literature, the FH, an uncommon presentation of the temporal bone, was found to have a prevalence of 12.3% in the patient sample studied by Liu et al. [15]. In our study, the presence of foramen Huschke was found to be 33.3% on the right side and 23.8% on the left side in patients with temporomandibular disorder. Few studies have explored the synchronization between TMJ disorders and the presence of FH, and there is a lack of research on the relationship between FH and TMJ [2, 16]. Our study found that the FH in patients with TMD was 2.11 ± 0.44 mm, underscoring the clinical significance of this anatomical variation. This indicated that the length of the FH was higher in the TMD group than in the control group. This anatomical variation has significant therapeutic consequences since a larger FH correlates with a heightened risk of TMJ herniation into the EAC. This herniation may lead to symptoms such as otalgia, otorrhea, and discomfort or pain during mastication, commonly observed in individuals with TMD.

The discovery of an enlarged FH in the TMD cohort indicates that FH morphology may affect the pathophysiology of TMD by modifying the structural and functional interactions between the TMJ and adjacent anatomical entities [17]. These findings underscore the significance of evaluating familial history aspects during the clinical assessment of patients with TMD, particularly in those exhibiting concomitant ear‐related symptoms. Identifying these structural variances could enhance the capacity to anticipate susceptibility to TMJ‐related disorders, refining diagnostic accuracy and facilitating more tailored treatment approaches. Subsequent studies should examine the mechanical ramifications of FH enlargement to enhance comprehension of its role in the initiation and advancement of TMD.

Furthermore, FH and pneumatized TMD osseous components can complicate clinical and surgical procedures involving the temporal bone region. Identifying these variations in imaging examinations is crucial [16]. For instance, Ertugrul [16] reported that using TMD arthroscopy with endoscopes smaller than 3 mm in diameter can potentially result in ear damage or TMD perforation.

In the literature review, only one publication addressed the relationship between the FH and TMJ positions and shapes [6]. Contrary to that publication's findings, our study found no relationship between condyle shape and the FH .

**TABLE 2:** Relationship between condyle shape and position with TMD and control group.

		Group		*p*
		TMD	Control	
Condyle shape	Oval	(42.2%) 57_a_	(57.8%) 78_a_	0.454
	Straight	(38.4%) 111_a_	(61.6%) 178_a_	0.675
Condyle position	Rear	(81.6%) 31_a_	(18.4%) 7_b_	0.000
	Top‐rear	(33.3%) 33_a_	(66.7%) 66_a_	
	Intracranial‐cranial	(100%) 33_a_	0_b_	
	Uncanged with no evidence of displacement	(28%) 71_a_	(72%) 183_b_	

*Note:* Test: *x* ^2^, *p* < 0.05, different subscript letters indicate significance.

Our findings indicate a higher incidence of these anatomical features in patients with TMJ, suggesting that these variations play a crucial role in the etiology and manifestation of TMJ symptoms. SO PTFs and larger FHs in patients with TMJ highlight the importance of thorough imaging and anatomical assessment in diagnosing and managing TMJ. These insights contribute to a better understanding of the underlying causes of TMJ and emphasize the need for tailored diagnostic and therapeutic strategies that consider these anatomical differences. This developmental defect can also lead to middle ear involvement, spreading the tumor or infection from the EAC to the infratemporal fossa. FH may cause injury to the external and middle ear structures during middle ear arthroscopy. Therefore, it is recommended to exercise caution in this area during surgical procedures. Future research should focus on further integrating advanced imaging techniques and clinical correlations to elucidate these variations' impact on TMJ, ultimately enhancing patient care and treatment outcomes. Future research should focus on longitudinal studies that track the development of TMD symptoms in patients with identified FH and PTF variations over time. Such studies can help establish causal relationships and provide insights into the progression of TMD in relation to these anatomical features.

A limitation of our study is the need for more information regarding tinnitus and MRI images for the patients. Further investigations, including detailed soft tissue analysis or clinical correlation with internal derangement, would be beneficial in elucidating the relationship between these structures and TMD more comprehensively. Future studies should incorporate a broader range of diagnostic tools and patient symptoms to provide a more holistic understanding of the implications of FH and PTF in TMJ.

## Author Contributions

**Hacer Eberliköse:** writing – review and editing, original draft, visualisation, validation, supervision, methodology, investigation, data curation, conceptualization. **Berrin Tuğtağ Demir:** writing – review and editing, methodology, investigation, formal analysis, conceptualization. **Raha Akbarihamed:** validation, reviewing. **Hakan Alpay Karasu:** writing – review and editing, conceptualization.

## Ethics Statement

This study was approved by the ethics committee of Ankara Medipol University (ethical no. 111).

## Conflicts of Interest

The authors declare no conflicts of interest.

## Data Availability

The data that support the findings of this study are available on request from the corresponding author. The data are not publicly available due to privacy or ethical restriction.
